# Prevalence and Phenotypic Characterization of Antibiotic-resistant *Enterococcus Species* Isolated from Chicken Faecal Samples in Accra, Ghana

**DOI:** 10.4314/ejhs.v34i4.2

**Published:** 2024-07

**Authors:** Philip Asumang, Frank Amoakohene, Collins Amponsah, Frank Kwasikumah, Emmanuel U Osisiogu, Israel M Attipoe

**Affiliations:** 1 Department of Science, Seventh-Day Adventist College of Education, Agona – Ashanti, Ghana; 2 Department of Biomedical Laboratory Sciences, School of Allied Health Sciences, University for Development Studies, Ghana; 3 Department of Medical Laboratory, Danpong Medical Centre, Accra, Ghana; 4 Department of Science Laboratory Technology, Dr. Hilla Limann Technical University, Wa, Ghana; 5 Department of Infectious Disease, School of Allied Health Sciences, University for Development Studies, Ghana

**Keywords:** Antibiotic resistance, multi-drug resistance, Enterococcus species, Antibiotics

## Abstract

**Background:**

Enterococci are bacteria found naturally in the gastrointestinal tract of both chickens and humans, serving as a commensal bacterium. These opportunistic pathogens are recognized for their involvement in human diseases like urinary tract infections (UTIs), endocarditis, and sepsis. Infection sources encompass food, hospital environments, and animals, particularly chickens. Their increasing resistance to multiple drugs poses a growing concern for public health

This study aimed to isolate the enterococcus species and to determine their antibiotic susceptibility profiles.

**Method:**

Swab samples of gut content from poultry in specific slaughterhouses located within selected markets in Accra were collected, cultured on MacConkey agar No.2, and incubated overnight for growth. Colonies suspected to be Enterococcus species were subjected to biochemical testing, and confirmed colonies underwent antibiotic testing against commonly used medications for bacterial infections. The Kirby-Bauer disk diffusion method was used to assess the antibiotic susceptibility of the recovered isolates.

**Results:**

Among the 160 samples examined, 97 (61%) were found to be contaminated with Enterococcus species. Each of the isolates displayed resistance to a minimum of three antibiotic classes tested in the study. Notably, high levels of resistance were observed for specific antibiotics, including penicillin (91.75%), vancomycin (87.63%), and tetracycline (80.41).

**Conclusion:**

The findings of this study revealed a high prevalence of multi-drug resistant Enterococcus species isolated from chicken rectal swab samples collected from three selected markets in Accra, Ghana. All the isolates exhibited resistance to at least three classes of antibiotics tested

## Introduction

*Enterococci*, typically considered harmless commensal microorganisms, are part of the natural flora in the intestinal tract, oral cavity, and genitourinary tract of both humans and animals. Traditionally referred to as ‘Faecal Streptococci,’ these bacteria were once seen as potential human pathogens able to cause various infections. However, in recent years, they have evolved into significant nosocomial pathogens with increased drug resistance, contributing substantially to patient morbidity and mortality (1). Their ability to survive and spread in hospital environments is primarily due to their intrinsic resistance to commonly used antibiotics. Additionally, their capacity to acquire resistance, either through mutation or the transfer of mobile genetic elements containing resistance genes and virulence factors, is a key factor (2). *Enterococci* can cause severe infections, including endocarditis, bacteremia, meningitis, intra-abdominal and pelvic infections, and burn and surgical site wound infections in both immune-competent and immune-compromised individuals. They pose a particular risk for infections in catheters, and implanted medical devices in critically ill patients, and can cause late-onset sepsis, pneumonia, and meningitis in neonates (3). According to the 2006-2007 report from the Centre for Disease Control and Prevention (CDC), *Enterococci* account for approximately 12% of healthcare-associated infections (HAI) and rank as the third most common multi-drug resistant pathogen causing HAI (4). The emergence of Vancomycin-resistant Enterococci (VRE) has been reported globally, with increasing incidences in various countries like Australia, Canada, Germany, Malaysia, Spain, and the United States (5). Besides colonizing humans and inhabiting animal reservoirs and the environment, VRE is a potential contributor to the acquisition and dissemination of antibiotic resistance determinants (6).

The Canadian Antimicrobial Resistance Surveillance System (CARSS) reports nearly four times greater antimicrobial use in animal husbandry compared to human use (7), contributing to resistance development in bacteria circulating in animal reservoirs. Poultry, a major animal reservoir, demonstrates increasing resistance over time, as evidenced by studies showing high resistance rates in enterococci isolates recovered from poultry litter samples (8). Studies also indicate a high likelihood of multidrug-resistant enterococci isolated from animal Faecal samples transferring their resistant traits to enterococci in humans, posing a significant threat to public health (9,10), these findings highlight poultry and their environment as suitable niches for enterococci antimicrobial resistance development and potential transfer of resistant traits to other bacterial species in humans or the environment. The alarming rate of antibiotic resistance emergence and dissemination poses a significant threat to both human and animal health (11). Heavy antibiotic use in poultry is identified as a major driver behind this resistance problem. The proliferation of antibiotic-resistant bacteria in food animals presents a substantial threat to human health (12). In humans, antibiotic-resistant infections lead to prolonged morbidity, increased mortality, and the spread of resistant pathogens. Prolonged hospitalization and the use of last-generation antibiotics also result in inflated patient hospital bills (13). This antibiotic resistance issue is particularly severe in developing countries with limited resources and ineffective check and surveillance mechanisms for antibiotic usage (14,15). The colonization of antibiotic-resistant *Enterococcus species* in poultry poses a significant risk to human health due to the potential for zoonotic transmission. These bacteria can be transmitted to humans through direct contact with infected birds or their faecal matter, as well as through the consumption of contaminated poultry products, such as undercooked meat or eggs. Cross-contamination can also occur during food preparation, where utensils, surfaces, or hands that have come into contact with raw poultry products can transfer the bacteria to other foods. Furthermore, the use of poultry manure as fertilizer in crop production can lead to the introduction of these resistant bacteria into the wider food chain.

The widespread use of antibiotics in poultry farming, often for growth promotion and disease prevention, plays a crucial role in driving the development and dissemination of antibiotic resistance. Subtherapeutic doses of antibiotics are frequently administered to poultry, creating an environment conducive to the emergence and persistence of resistant strains. Additionally, improper dosing or extended use of antibiotics can exacerbate the problem by exerting selective pressure on bacterial populations, favouring the survival and proliferation of resistant organisms. Consequently, poultry can serve as reservoirs for antibiotic-resistant bacteria, posing a potential threat to public health through the aforementioned transmission routes. Therefore, this study aimed to isolate the enterococcus species and to determine their antibiotic susceptibility profiles.”

## Methods

**Study design**: A cross-sectional study was employed to determine the prevalence of antibiotic-resistant *Enterococcus species* in three different poultry markets. Faecal samples were collected from selected markets and analyzed from February 2023 to June 2023.

**Study sites**: Samples of chicken gut content were obtained from one slaughterhouse in each of the selected markets located within the Accra; Makola, Agbogbloshie, and Kaneshie markets. These markets were selected because they are the major markets in Accra, with numerous sellers and customers from Accra and beyond. It is patronized by both consumers and retail sellers.

**Sample collection**: Swab samples of chicken gut content were taken from slaughterhouses located in the selected markets after permission was obtained from the vendors. Strict aseptic techniques were followed during the slaughter and sample collection process to prevent contamination. The samples were taken from the intestines of freshly slaughtered healthy chickens and placed in sterile stool collection containers. The stool containers were then placed in an ice chest containing ice and transported immediately to the laboratory for processing. One hundred and sixty (160) stool samples were obtained from all the market centers based on the availability of samples and willingness to participate in this study.

**Laboratory procedure**: On arrival to the laboratory, the stool samples were streaked onto Blood agar and MacConkey agar No.2 with bile salt (Biomark, India) and incubated for 24 hours at 37°C. The growth of lactose fermenters was detected by the presence of small, intensely colored red-purple colonies. Preliminary tests for identification such as Gram stain, catalase test, and motility test were performed on the selected isolates. Catalase-negative, and Gram-positive cocci in pairs and short chains were selected and processed further. Pure *Enterococcus* colonies were inoculated into cryotubes and stored for future use. Mixed growth/contaminated colonies were sub-cultured on MacConkey agar (Biomark, India) to obtain pure colonies before storage.

**Bile esculin agar (BAE)**: The suspected isolates were then inoculated onto Bile esculin agar (containing 40% bile), and incubated aerobically at 37°C overnight. The next day the isolates showing black discolouration of the medium due to hydrolysis of esculin to esculetin were identified as BEA positive. After studying the colonial morphology of each isolate obtained from blood agar, MacConkey agar No.2, and bile esculin agar, the isolates that were non-hemolytic on blood agar, showed magenta pink-colored tiny colonies on MacConkey agar No.2 and BEA positive were selected for further biochemical reactions.

**Heat tolerance test**: The suspected Enterococcal isolates were tested for heat tolerance by inoculating them into Brain Heart Infusion (BHI) broth and incubating them at 60°C for 30 minutes in a water bath. Subcultures from the broth were done on blood agar and MacConkey agar No.2 before incubation and at intervals of 10 minutes, 20minutes and 30 minutes after incubation. The isolates showing growth before and after 30 minutes of incubation at 60°C were taken as heat-tolerant Enterococcal isolates.

**Salt tolerance**: Salt tolerant property of the suspected Enterococcal isolates was tested by inoculating 2 to 3 identical colonies of suspected isolates into a tube containing nutrient broth with 6.5% sodium chloride and incubated at 37°C for 24-72 hours. 1% bromocresol purple was added as an indicator to detect yellow discoloration on growth. The broth showing turbidity with or without yellow discoloration was taken as a positive reaction and was confirmed by subculturing the broth on blood agar and MacConkey agar No.2. Salt tolerant, BEA-positive isolates, which were able to grow on MacConkey agar No.2 and at temperatures of > 45°C were identified as Enterococci.

**Antibiotic sensitivity testing**: Antimicrobial susceptibility of isolated *Enterococcus species* was carried out using the Kirby-Bauer method of disk diffusion (16). The following antimicrobial drugs (Biomark, India) were used: lincomycin (2 µg), erythromycin (15 µg), vancomycin (2 µg), cotrimoxazole (25 µg), tetracycline (30 µg), gentamicin (10 µg), cephalexin (30 µg), penicillin (10 µg), ciprofloxacin (5 µg), cloxacillin (10 µg) and levofloxacin (5 µg). Most of these antibiotics are commonly used in Ghana for the treatment of infections (17,18). Pure colonies from each confirmed, Enterococcus isolate were suspended in sterile saline to obtain turbidity equivalent to 0.5% Mcfarland standard. The suspension was diluted with 9 parts of saline to obtain a concentration, equivalent to 1 x 10^7^ colony-forming unit and then inoculated on a Mueller-Hinton agar (Biomark, India) plate, using a sterile cotton-tipped swab. The inoculated plate was allowed to dry for 10 minutes. The selected antibiotic-impregnated disks were applied aseptically on the inoculated Mueller-Hinton agar plate. The plate was then incubated aerobically at 37°C for 24 hours. After incubation, the zone of inhibition of each of the antibiotics was measured and interpreted as per the European Committee on Antimicrobial Susceptibility Testing (19).

## Results

**Figure 1:**
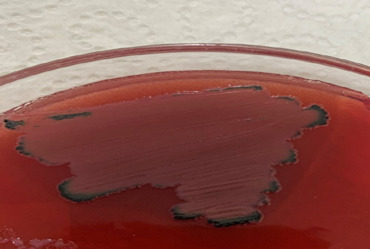
Photograph of Enterococcus species on Bile esculin agar

A total of hundred and sixty (160) swabs of chicken gut content was collected; seventy-five (75) were from the Makola market, fifty (50) from the Agbogbloshie market, and thirty-five (35) from the Kaneshie market. Table 1 shows the different sampling sites, the number of samples, and several bacteria isolates as well as the resistance patterns observed. Ninety-seven (97) isolates were obtained from the 160 faecal swab samples, indicating a prevalence of 61%. The isolated colonies displaying characteristic black discoloration on bile esculin agar, indicating Enterococcus species, are shown in Figure 1.

**Table 1:** Distribution and Antibiotic Resistance Profiles of Enterococcal Isolates

Markets	No. of Samples	No. of Isolates	Number of Resistant Isolates										

			LIN	TET	COT	GEN	VAN	ERY	CLO	PR	CIP	PEN	LEV
*Agbogbloshie*	50	36	0	25	9	4	28	7	34	19	5	32	3
*Makola*	75	49	0	43	8	4	45	9	20	10	3	46	1
*Kaneshie*	35	12	1	10	3	0	12	6	12	5	1	11	1

KEY: LIN = Lincomycin TET = Tetracycline COT = Co-trimoxazole GEN = Gentamicin VAN = Vancomycin ERY = Erythromycin CLO = Cloxacillin PR = Cephalexin CIP = Ciprofloxacin PEN = Penicillin LEV = Levofloxacillin

**Figure 2:**
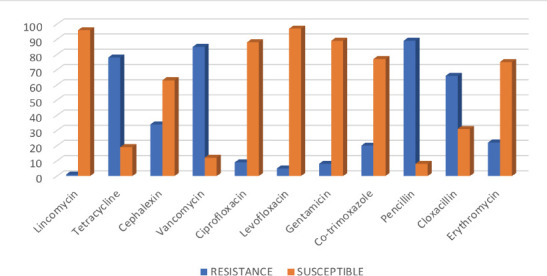
Antibiotic Resistance and Susceptibility of Enterococcus Isolates

**Antimicrobial resistance pattern of Enterococcal isolates**: The results of antimicrobial testing conducted on all ninety-seven (97) isolates revealed an alarming pattern of resistance. A substantial majority (91.6%) of the isolates displayed resistance to penicillin, indicating a widespread phenomenon. Notably, every isolate demonstrated resistance to the entire spectrum of antibiotics tested (Figure 2).

In particular, vancomycin exhibited a significantly high resistance rate of 87.6%, indicating a significant decrease in its efficacy. Following closely were tetracycline with a resistance rate of 80.4%, and cloxacillin at 68% all of which displayed substantial levels of resistance. Cephalexin and erythromycin showed lower but still significant resistance levels at 35.1% and 22.7%, respectively. On a relatively positive note, some antibiotics, including lincomycin, levofloxacin, gentamicin, and ciprofloxacin, demonstrated lower resistance levels, ranging from 0% to 10%.

Resistance to a penicillin (91.6%), tetracycline (80.41%), and vancomycin (87.6%) were the highest while lincomycin was the least resisted antibiotic i.e., 1% as shown in Figure 2.

**Multi-drug resistance in the *Enterococcus* isolates**: All of the isolates were resistant to three or more classes of antibiotics (Table 2). A few (3.1%) of the isolates were found to be resistant to nine classes of antibiotics tested. Most of the isolates (65%) were resistant to more than four antibiotics.

**Table 2:** Multiple antibiotic drug resistance in *Enterococcus* isolates

No. drug classes*	Resistant

	N (%)
3	3(3.1)
4	27(27.8)
5	33(34.0)
6	19(19.6)
7	8(8.3)
8	4(4.1)
9	3 (3.1)

* Number of drug classes to which isolates were resistant

## Discussions

Although antibiotics have played a crucial role in reducing illness and fatalities associated with infectious diseases in humans and animals (20), their widespread use, especially in poultry, can lead to an increase in drug resistance among both beneficial and pathogenic bacteria (21,22). Enterococci have a wide array of resistant mechanisms that promote survival in human and hospital environments, including modification of drug targets, inactivation of therapeutic agents, overexpression of efflux pumps, and a sophisticated cell envelope adaptive response. Enterococci adaptation mechanisms vary for acquiring resistant determinants mainly via conjugative plasmid and transposons. Since enterococci can resist harsh environmental conditions, enterococci facilitate survival and spreading of resistance in the environment (23).

The results of this study show a high prevalence of multidrug resistance in *Enterococcus* isolates. There is 100% multi-resistance to three or more classes of antibiotics. This is in agreement with the results of a study on *Enterococcus* from a chicken stool by Iweriebor (24) that reported a very high multi-resistance to antibiotics tested with vancomycin, streptomycin and cloxacillin having 100 % resistance respectively. The high resistance to antibiotics in poultry is largely influenced by the antibiotic use in poultry as well as poor sanitation and overcrowding in poultry coops (25,26). This explains the high prevalence of multidrug-resistant *Enterococcus* isolates in chickens as shown in table 4 of this study.

The high resistance rates were observed against penicillin, vancomycin, tetracycline, co-trimoxazole and cloxacillin. Penicillin, tetracycline, and co-trimoxazole have been on the Ghanaian markets for a significantly longer period than antibiotics like ciprofloxacin, lincomycin, and levofloxacin, which were introduced more recently (27) hence its use is expected to be dominant in animal farming. Furthermore, they have been reported to be highly resisted by several bacteria for the past years (28). Cloxacillin has been on the Ghanaian markets since 2002, which is relatively too recent to expect such a high resistance in this study (29). High resistance to vancomycin and cloxacillin could be due to the transfer of resistance as attributed to an increased multidrug resistance rate (30). Moreover, the production of REG3 gamma leads to the killing of Gram-positive bacteria (including VRE) thus working as a part of the mucosal innate immune defense mechanism. However, using broad-spectrum antibiotics like vancomycin can reduce REG3 gamma levels, which can allow antibiotic-resistant bacteria like VRE to colonize and cause infections (31).

Among the antibiotics tested, *Enterococcus species* exhibited the lowest resistance towards lincomycin, levofloxacin, and ciprofloxacin. Ciprofloxacin, Lincomycin, and Levofloxacin have been on the market for a shorter period as compared to Penicillin and Tetracycline (27). This explains the high susceptibility of *Enterococcus species* to these antibiotics as time is required for resistance development. Moreover, they are very expensive to purchase and that might have discouraged their proliferative use in animal farming (27). Ciprofloxacin, Lincomycin, and Levofloxacin are among the most active antibiotics as used in this study. However, resistance to ciprofloxacin and other fluoroquinolones have been linked with the up-regulation of efflux pumps, which transport fluoroquinolone drugs out of the bacterial cell (32). Secondly, mutations in the quinolone resistance determining genes follow a common mechanism, suggesting possible multi-quinolone-drug resistance by one resistant trait (33).

The presence of antibiotic-resistant *Enterococcus species* and other bacteria in food animals raises concerns regarding human health. These animals serve as potential reservoirs of these bacteria, posing a risk of infection to humans, particularly those working with poultry and in slaughterhouses. While many Ghanaians extensively cook their meat before consumption, the utensils and surfaces used in cooking can serve as mediums for transferring these bacteria to other uncooked food items like vegetables. Additionally, consuming fresh, uncooked eggs poses a potential risk of infection. The eggshell, often carrying these bacteria, can contaminate its contents during processing. Furthermore, the use of chicken droppings, which harbour these enteric bacteria, as organic fertilizer in vegetable farming can lead to crop contamination, ultimately exposing consumers to these bacteria. In light of the above, there is a need for judicious use of these antibiotics to minimize the rate of their resistance in the country.
